# Reduced Local Tumor Progression After Thermal Ablation During Atezolizumab Plus Bevacizumab Treatment for Hepatocellular Carcinoma

**DOI:** 10.3390/cancers18111800

**Published:** 2026-06-01

**Authors:** Tasuku Nakabori, Kaori Mukai, Taku Miyanaga, Hiroki Takiyama, Keita Sekiya, Takumi Kinomoto, Takanori Masumoto, Jun Murata, Makiko Urabe, Yugo Kai, Ryoji Takada, Toshitaka Morishima, Minoru Shigekawa, Kazuyoshi Ohkawa

**Affiliations:** 1 Department of Hepatobiliary and Pancreatic Oncology, Osaka International Cancer Institute, Osaka 541-8567, Japanmakiko.urabe@oici.jp (M.U.); ryoji.takada@oici.jp (R.T.); minoru.shigekawa@oici.jp (M.S.);; 2 Cancer Control Center, Osaka International Cancer Institute, Osaka 541-8567, Japan

**Keywords:** ablation, local tumor progression, atezolizumab plus bevacizumab, hepatocellular carcinoma, immunotherapy

## Abstract

**Background/Objectives**: Although thermal ablation is an established curative treatment for early-stage hepatocellular carcinoma (HCC), local tumor progression (LTP) after ablation remains a clinically important issue. In advanced-stage HCC treated with immunotherapy, ablation is increasingly performed concurrently with atezolizumab plus bevacizumab (atezo/bev). However, evidence regarding its clinical outcome is limited. This study aimed to evaluate the association between concurrent atezo/bev and LTP after ablation. **Methods**: This retrospective study included 467 ablated tumors from 376 treatment courses. Tumors were classified into two groups according to atezo/bev administration status. The cumulative incidence of LTP was compared between the two groups. Factors associated with LTP and ablation-related complications were also assessed. **Results**: Twenty-five tumors were ablated during atezo/bev treatment in 17 treatment courses (ablation + atezo/bev group), whereas 442 tumors were ablated without concurrent atezo/bev in 359 treatment courses (ablation-alone group). The cumulative incidence of LTP was significantly lower in the ablation + atezo/bev group than in the ablation-alone group (*p* = 0.031). In the multivariable analysis, ongoing atezo/bev treatment was independently associated with a reduced risk of LTP (hazard ratio, 0.124; *p* = 0.015). No significant differences in ablation procedure-related complications were observed between the two groups. **Conclusions**: Thermal ablation during atezo/bev treatment was associated with a reduced risk of LTP without an apparent increase in procedure-related complications. These findings suggest that concurrent administration of atezo/bev may be associated with improved local tumor control after ablation, supporting the feasibility of ablation as part of multidisciplinary treatment strategies for advanced-stage HCC.

## Simple Summary

Although thermal ablation is an established curative treatment for early-stage hepatocellular carcinoma (HCC), local tumor progression (LTP) after ablation remains a clinically important concern. In advanced-stage HCC treated with immunotherapy, ablation is increasingly performed concurrently with atezolizumab plus bevacizumab (atezo/bev). This study investigated whether thermal ablation performed during atezo/bev treatment influences LTP in HCC. A total of 467 ablated tumors were retrospectively analyzed and categorized according to concurrent atezo/bev use. The cumulative incidence of LTP was significantly lower in the ablation + atezo/bev group than in the ablation-alone group. Multivariate analysis demonstrated that ongoing atezo/bev treatment was independently associated with a reduced risk of LTP. The incidence of ablation-related complications was comparable between the two groups. These findings suggest that concurrent atezo/bev may be associated with improved local tumor control after ablation without compromising procedural safety. Thus, incorporating ablation during atezo/bev treatment may be feasible for the multidisciplinary management of advanced-stage HCC.

## 1. Introduction

Hepatocellular carcinoma (HCC) is a leading cause of cancer-related mortality worldwide [1]. For early-stage HCC, surgical resection and thermal ablation, most commonly radiofrequency ablation (RFA) or microwave ablation (MWA), are established curative treatment options [2]. However, local tumor progression (LTP) after ablation remains a clinically important issue, necessitating repeated interventions and negatively affecting survival outcomes [3,4]. Therefore, it is essential to identify the factors associated with LTP. Previous studies have reported several risk factors for LTP, which can be classified as host-related factors (e.g., impaired hepatic reserve), tumor-related factors (e.g., larger tumor size and elevated serum tumor markers), and procedure-related factors (e.g., insufficient ablative margins) [5,6,7,8].

The therapeutic landscape of advanced-stage HCC has evolved substantially with the introduction of combination immunotherapies, including atezolizumab plus bevacizumab (atezo/bev), tremelimumab plus durvalumab, and nivolumab plus ipilimumab, which are the current standard first-line systemic treatments [9,10,11,12,13,14]. In the era of immunotherapy, the role of locoregional treatment (LRT) has expanded beyond conventional indications, as accumulating evidence suggests that its combination with systemic therapy may improve clinical outcomes [15,16,17]. Consequently, ablation is increasingly performed concurrently with systemic therapy, including atezo/bev. However, evidence regarding its oncological effectiveness remains limited. Therefore, this study aimed to evaluate the clinical outcomes of thermal ablation performed during atezo/bev treatment, focusing on LTP after ablation.

## 2. Materials and Methods

### 2.1. Study Population

Clinical data from consecutive patients with HCC who underwent thermal ablation with RFA or MWA at the Osaka International Cancer Institute between May 2021 and July 2025 were retrospectively collected. HCC was diagnosed according to the American Association for the Study of Liver Diseases criteria [18,19]. The exclusion criteria were: (1) HCC exceeding the Milan criteria [20]; (2) post-ablation follow-up <2 months; (3) lack of contrast-enhanced liver imaging; (4) ablation combined with percutaneous ethanol injection therapy or transarterial chemoembolization; (5) ablation combined with bile duct cooling using an endoscopic nasobiliary drainage tube [21]; (6) use of investigational agents or lenvatinib [22] during the peri-ablative period; (7) insufficient ablative margin, except for areas adjacent to blood vessels or bile ducts; and (8) HCC recurrence after atezo/bev discontinuation due to a complete response (CR). All included tumors met the Milan criteria at the time of ablation because tumors exceeding the Milan criteria were excluded according to the study design. Tumors were classified into two groups based on the atezo/bev administration status: ablation + atezo/bev and ablation-alone groups. Liver function was assessed using the Child–Pugh classification and albumin–bilirubin (ALBI) grade. Previous HCC treatment history was evaluated based on whether tumors were primary or recurrent at the time of ablation. Primary cases were defined as HCC diagnosed for the first time. The date of ablation was defined as the start of the follow-up, which was censored on 31 December 2025. The median observation period for ablated tumors was 560 days (range, 70–1667 days). This study was conducted in accordance with the Declaration of Helsinki and approved by the Institutional Review Board of Osaka International Cancer Institute (approval number 25197).

### 2.2. Thermal Ablation Technique

Thermal ablation was carried out using RFA or MWA systems (including Arfa RF ablation system; Japan Lifeline, Tokyo, Japan; and VIVA RF system; STAR Med, Gyeonggi, Korea for RFA, and Emprint ablation system; Covidien/Medtronic, Minneapolis, MN, USA; and Mimapro; Mima-pro Scientific Inc., Nantong, China for MWA), with the specific modality and device selected according to the operator’s clinical judgment.

### 2.3. Definition of a High-Risk Location

Based on previously reported criteria [23], tumors located in close proximity (≤5 mm) to major anatomical structures were categorized as high-risk. These structures included the first- or second-order branches of the portal vein or bile duct, major hepatic veins, and the inferior vena cava.

Tumors located within 5 mm of the extrahepatic organs, such as the gallbladder or gastrointestinal tract, were initially classified as high-risk but were excluded from this category if an adequate safety margin was achieved by artificial pleural effusion or ascites before ablation.

### 2.4. Atezo/Bev Treatment

Atezolizumab (1200 mg) and bevacizumab (15 mg/kg) were administered according to institutional clinical practice and previously reported criteria for unresectable HCC [24,25]. Patients with clear contraindications to immunotherapy or bevacizumab, including uncontrolled bleeding risk, severe autoimmune disease, and poor general condition, were not considered candidates for atezo/bev treatment. Ablation was performed concurrently with atezo/bev for two purposes: (1) eradication of residual viable tumors after marked tumor shrinkage to achieve CR, referred to as curative conversion therapy (ABC conversion) [26,27], and (2) salvage treatment for progressive disease (PD) targeting oligo-drug-resistant lesions, followed by continuation of atezo/bev (PD salvage) [24,25]. The indications for ablation in ABC conversion and PD salvage followed previously reported criteria [24,25].

### 2.5. Evaluation of Treatment Effect and Safety

Radiological response was determined using either contrast-enhanced computed tomography (CT) or gadolinium–ethoxybenzyl diethylenetriamine pentaacetic acid-enhanced magnetic resonance imaging (EOB-MRI). Ablative margins were classified using three-dimensional imaging registration, as previously described [6], as complete ablation except for areas adjacent to vessels or bile ducts (Rvf1′), <5 mm (Rvf2), or ≥5 mm (Rvf3) encompassing the entire tumor. When a residual tumor or an insufficient ablative margin was identified, additional ablation was performed and considered part of the same treatment course. LTP was defined as tumor growth within or adjacent to the ablation zone. Tumors were censored when another intrahepatic recurrence developed adjacent to the ablation scar because an accurate evaluation of LTP was no longer feasible, as previously reported [6]. Major complications were defined as events resulting in substantial morbidity, hospital admission, or prolonged hospital stay [28].

### 2.6. Follow-Up Surveillance

Follow-up surveillance with contrast-enhanced CT or EOB-MRI and blood tests, including tumor markers (α-fetoprotein [AFP] and des-γ-carboxy prothrombin [DCP]), was performed every 3 months. Additional imaging or blood tests were performed when at least one tumor marker was elevated.

During the atezo/bev treatment, contrast-enhanced CT or EOB-MRI was performed every 6–9 weeks.

### 2.7. Statistical Methods and Analysis

Continuous variables are summarized as medians with ranges and were compared using the Mann–Whitney U test. Categorical data are described as frequencies and were analyzed using either the Pearson’s chi-square test or Fisher’s exact test, as appropriate. Cumulative incidence of LTP was estimated using the Kaplan–Meier method and differences between groups were assessed with the log-rank test. Hazard ratios (HRs) and corresponding 95% confidence intervals (CIs) were derived from Cox proportional hazards models. The proportional hazards assumption was assessed using Schoenfeld residual tests in the corresponding conventional Cox proportional hazards models. Given the limited number of LTP events, multivariate analysis was performed using Firth’s penalized likelihood Cox regression [29,30] to reduce sparse-data bias and improve estimation stability. Variables with *p* < 0.1 in the univariate analysis were included in the multivariate model. Statistical significance was assessed using profile likelihood-based CIs and likelihood-ratio tests. To address potential intra-patient correlation among multiple tumors treated in the same patient, additional analyses were also performed at the treatment-course level. A *p*-value < 0.05 was considered statistically significant. All analyses were performed using EZR (Saitama Medical Center, Jichi Medical University, Saitama, Japan), a graphical interface for R Commander (version 1.61) on Windows [31].

## 3. Results

### 3.1. Characteristics of Ablated Tumors and Treatment Courses

**Figure 1:**
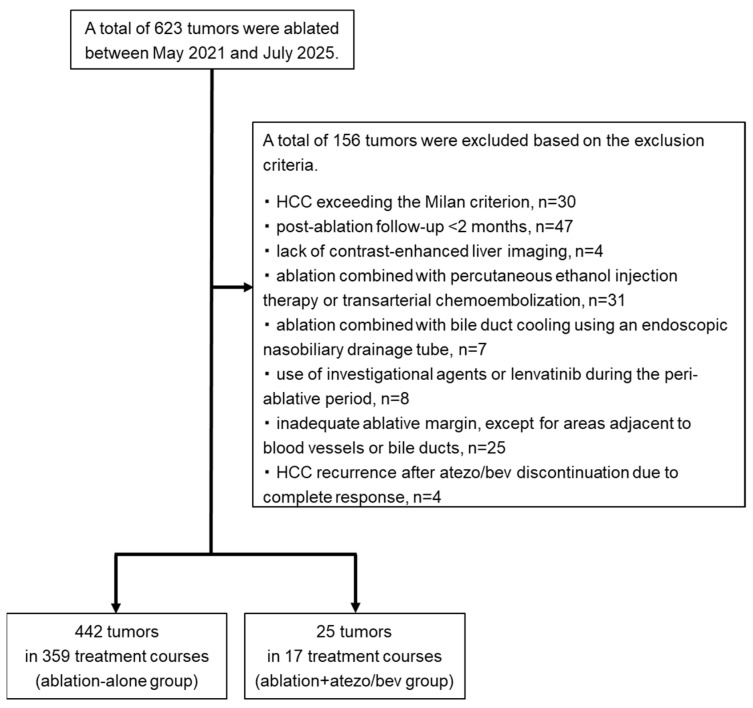
Flowchart of the study. atezo/bev, atezolizumab plus bevacizumab.

A total of 467 tumors from 376 treatment courses were included in this study. The study flowchart is presented in Figure 1. The characteristics of ablated tumors and treatment courses are summarized in Table 1 and Table 2, respectively. Ablation was performed during atezo/bev treatment in 25 tumors across 17 treatment courses (ablation + atezo/bev group), whereas 442 tumors across 359 treatment courses underwent ablation without concurrent atezo/bev (ablation-alone group). In the ablation + atezo/bev group, eight tumors underwent ablation for ABC conversion to achieve CR, whereas 17 atezo/bev-resistant tumors were treated with ablation as PD salvage. The characteristics of ablated tumors and treatment courses in the ABC conversion and PD salvage subgroups are summarized in Tables S1 and S2, respectively.

There were no significant differences for ablated tumors between the ablation-alone and ablation + atezo/bev groups in terms of tumor diameter, frequency of high-risk location, or ablative margin status (Table 1). The median observation period for the ablated tumors was longer in the ablation + atezo/bev group (738 days [range, 178–1488]) than in the ablation-alone group (541 days [range, 70–1667]; *p* = 0.044).

Per treatment course, age, sex, proportion of primary HCC, hepatic reserve, ablation device, frequency of multiple tumor ablations, and use of ancillary procedures, including Sonazoid-enhanced imaging and artificial pleural effusion or ascites, did not differ significantly between the two groups (Table 2). Viral etiology was more prevalent in the ablation + atezo/bev group. Serum AFP levels at the time of treatment were comparable; however, serum DCP levels were significantly higher in the ablation + atezo/bev group than in the ablation-alone group.

Regarding the comparison between the ABC conversion and PD salvage subgroups, no significant differences were observed in variables related to the ablated tumors or treatment courses (Tables S1 and S2).

### 3.2. Comparison of LTP Between the Ablation-Alone and Ablation + Atezo/Bev Groups

**Figure 2:**
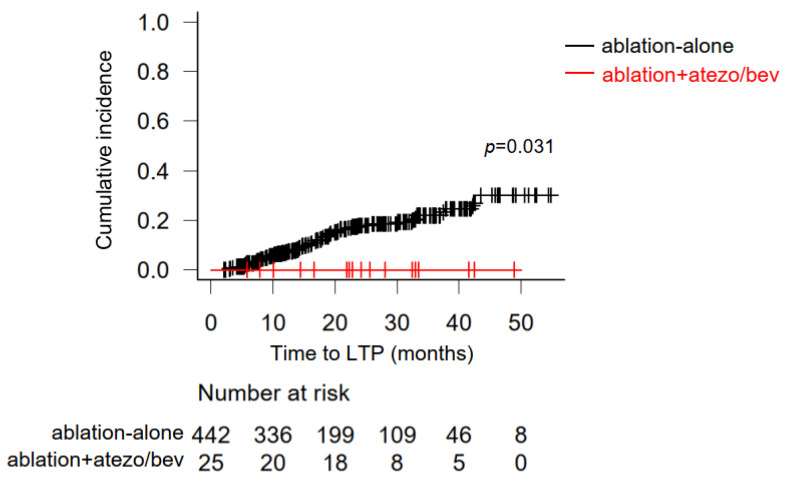
Cumulative incidence curves for LTP. Curves represent cumulative incidence estimated using the Kaplan–Meier method (1 − Kaplan–Meier estimates). atezo/bev, atezolizumab plus bevacizumab; LTP, local tumor progression.

Cumulative LTP curves for the two groups are shown in Figure 2 and Figure S1. The 1-year and 2-year cumulative LTP rates were 6.9% (95% CI: 4.8–9.9%) and 17.9% (95% CI: 13.9–22.9%), respectively, in the ablation-alone group, whereas no LTP events were observed in the ablation + atezo/bev group. The incidence of LTP was significantly lower in the ablation + atezo/bev group than in the ablation-alone group (*p* = 0.031).

### 3.3. Factors Associated with LTP After Ablation

The factors associated with LTP after ablation were evaluated (Table 3 and Table 4).

In the per-tumor univariate analysis, ongoing atezo/bev treatment was significantly associated with a reduced risk of LTP (HR, 0.121; 95% CI, 0.001–0.840; *p* = 0.027). In the multivariable Cox proportional hazards model, including high-risk location, ablative margin of Rvf1′, and ongoing atezo/bev treatment, ongoing atezo/bev treatment remained independently associated with a reduced risk of LTP (HR, 0.124; 95% CI, 0.001–0.859; *p* = 0.015).

In the per-treatment-course univariate analysis, serum AFP level ≥ 10 ng/mL (HR, 2.116; 95% CI, 1.251–3.581; *p* = 0.005) and ongoing atezo/bev treatment (HR, 0.128; 95% CI, 0.001–0.885; *p* = 0.032) were significantly associated with LTP. In the multivariable model including age ≥ 65 years, serum AFP level ≥ 10 ng/mL, and ongoing atezo/bev treatment, serum AFP level ≥ 10 ng/mL (HR, 2.197; 95% CI, 1.280–3.665; *p* = 0.005) and ongoing atezo/bev treatment (HR, 0.132; 95% CI, 0.001–0.913; *p* = 0.037) remained independently associated with LTP.

### 3.4. Comparison of Ablation Procedure-Related Complications Between the Ablation-Alone and Ablation + Atezo/Bev Groups

Ablation procedure-related complications are summarized in Table 5. No significant differences in the complication rates were observed between the two groups.

## 4. Discussion

Atezo/bev has demonstrated significant improvements in survival and tumor response and is now a standard first-line treatment in advanced-stage HCC. Bevacizumab-mediated vascular endothelial growth factor (VEGF) inhibition exerts antiangiogenic effects and modulates the immunosuppressive tumor microenvironment, thereby providing a rationale for combination strategies with LRTs [32]. Consequently, the integration of LRTs with atezo/bev has attracted increasing clinical interest, and ablation performed during ongoing atezo/bev treatment is expected to become more common. However, detailed analyses of clinical outcomes after ablation performed concurrently with atezo/bev, particularly LTP, remain scarce. Therefore, this study investigated the clinical significance of concurrent atezo/bev in terms of LTP.

In the present study, the cumulative incidence of LTP in the ablation-alone group was comparable to that reported previously (7.7–9.1% at 1 year and 16.4–30.4% at 2 years after ablation) [33,34]. In contrast, tumors ablated during atezo/bev treatment showed a significantly lower cumulative incidence of LTP. Multivariable Cox analysis further demonstrated that ongoing atezo/bev treatment was independently associated with a reduced risk of LTP, suggesting a potential role for concurrent atezo/bev in contributing to improved local tumor control after ablation therapy.

Ablation performed during atezo/bev served two clinical purposes: curative conversion (ABC conversion) and salvage treatment for PD (PD salvage). No LTP events were observed during the follow-up period in either the ABC conversion or PD salvage subgroup. In the ABC conversion setting, continued atezo/bev administration may favor suppression of LTP after ablation, because tumors remain sensitive to atezo/bev. On the other hand, PD salvage targets atezo/bev-resistant tumors, suggesting that alternative mechanisms may underlie the observed reduction in LTP. One possible explanation for this finding is the augmentation of antitumor immunity induced by LRT, as experimental studies have shown that LRT, including radiotherapy and thermal ablation, promotes tumor antigen release and subsequent immune activation, thereby enhancing systemic antitumor immune responses [35,36].

In addition to immune modulation associated with PD-L1 blockade by atezolizumab, VEGF inhibition by bevacizumab may also have contributed to improved local tumor control through tumor vascular normalization, enhanced immune cell infiltration, and suppression of residual microscopic tumor growth after ablation [37]. However, this hypothesis remains speculative because of the absence of direct immunological data in the present study. Thus, further investigation is warranted to clarify the interaction between ablation and atezo/bev treatment in this clinical setting.

Elevated serum AFP levels were associated with an increased risk of LTP, consistent with previous reports linking AFP elevation to aggressive microscopic features such as microvascular invasion and satellite nodules [38,39]. These occult peritumoral lesions may contribute to residual disease beyond the visible tumor margin, even after apparently complete ablation.

Serum DCP levels at the time of treatment were significantly higher in the ablation + atezo/bev group than in the ablation-alone group. It remains unclear whether this difference reflected more aggressive tumor biology [40]; the effects of VEGF inhibition by bevacizumab, which has been reported to induce hypoxia, which may enhance DCP production [41,42]; or the presence of residual non-ablated tumors in patients undergoing PD salvage treatment. However, serum DCP levels were not significantly associated with LTP in the Cox proportional hazards analyses.

No significant differences in ablation procedure-related complications were observed between the ablation-alone and ablation + atezo/bev groups. Although bevacizumab-mediated inhibition of the VEGF pathway raises concerns regarding bleeding and delayed wound healing [43,44], these adverse effects were not increased in the present cohort. These findings suggest that with appropriate patient selection and peri-procedural management, ablation can be safely performed concurrently with atezo/bev.

Recent real-world studies have demonstrated favorable safety and efficacy of sequential systemic therapies in the immunotherapy era, including regorafenib following immunotherapy failure and lenvatinib for patients who are unsuitable for immunotherapy [45,46]. In this paper, improved local tumor control achieved by ablation during atezo/bev treatment may contribute not only to eradication of resistant lesions but also to maintenance of overall treatment strategy continuity. Therefore, individualized multidisciplinary approaches integrating systemic therapies and LRTs according to tumor status and treatment response may become increasingly important in the management of unresectable HCC.

This study is subject to some limitations. First, the retrospective design may have introduced selection bias, as patients who underwent ablation during atezo/bev treatment, particularly those selected for ABC conversion, may have represented a clinically or biologically favorable subgroup with a certain degree of disease control or clinical stability before ablation. In addition, since some patients underwent ablation for multiple tumors, potential intra-patient correlation could not be completely excluded. Although similar findings were observed in both the per-tumor and per-treatment-course analyses, advanced statistical approaches, such as frailty models or cluster-adjusted analyses, were not applied due to the limited number of LTP events in the ablation + atezo/bev group. Second, the relatively small number of tumors ablated during atezo/bev treatment and the limited number of LTP events restricted our ability to perform detailed subgroup analyses according to treatment indication, such as ABC conversion and PD salvage. Moreover, although Schoenfeld residual tests showed no significant violations of the proportional hazards assumption and Firth’s penalized likelihood Cox regression was applied to mitigate estimation bias caused by the zero-event group, the resulting hazard ratio should still be interpreted with caution due to the substantial imbalance in sample size between the groups and sparse-event conditions. In addition, although no procedure-related complications were observed in the ablation + atezo/bev group, definitive conclusions regarding safety, particularly with respect to bevacizumab-related adverse events, could not be drawn. Third, this study primarily focused on LTP and did not assess other clinically relevant outcomes, such as recurrence-free survival, overall survival, or patterns of intrahepatic recurrence. Fourth, the observed reduction in LTP after ablation during immunotherapy should be interpreted specifically within the context of atezo/bev treatment. Therefore, these findings cannot be readily generalized to other immunotherapy regimens, such as tremelimumab plus durvalumab or nivolumab plus ipilimumab. Further validation in larger, prospective, multicenter studies and real-world multicenter analyses, including adjustment for key clinical variables using more sophisticated statistical approaches such as propensity score methods, is warranted. Moreover, evaluation of broader clinical endpoints will be necessary to more comprehensively assess the clinical impact of ablation during immunotherapy.

## 5. Conclusions

Ablation performed during atezo/bev treatment was associated with a lower incidence of LTP without an apparent increase in procedure-related complications. These findings suggest that concurrent administration of atezo/bev may be associated with improved local tumor control after ablation and that ablation performed under appropriate peri-procedural management could be considered a feasible LRT option within multidisciplinary strategies for unresectable HCC.

## Figures and Tables

**Table 1:** Characteristics of ablated tumors with and without concurrent treatment with atezolizumab plus bevacizumab.

		Concurrent Atezolizumab Plus Bevacizumab		
Variable	All Tumors (*n* = 467)	Without (*n* = 442)	With (*n* = 25)	*p*-Value
Tumor diameter, mm	15 (5–30)	15 (5–30)	17 (7–29)	0.125
Tumor location, C/A/P/M/L	7/174/149/41/96	6/165/139/39/93	1/9/10/2/3	0.485
High-risk location, yes/no	69/398	65/377	4/21	0.776
Ablative margin, Rvf1′/2/3	50/373/44	46/355/41	4/18/3	0.436

Continuous variables are presented as medians (ranges). A, anterior segment; C, caudate lobe; L, lateral segment; M, medial segment; P, posterior segment.

**Table 2:** Characteristics of treatment courses with and without concurrent administration of atezolizumab plus bevacizumab.

		Concurrent Atezolizumab Plus Bevacizumab		
Variable	All Courses (*n* = 376)	Without (*n* = 359)	With (*n* = 17)	*p*-Value
Age, years	75 (41–93)	75 (41–93)	73 (51–82)	0.388
Sex, male/female	299/77	283/76	16/1	0.216
Primary/Recurrent	77/299	76/283	1/16	0.215
Etiology, viral/non-viral	148/228	137/222	11/6	**0.040**
Child–Pugh grade A/B	348/28	331/28	17/0	0.627
ALBI score	−2.678 (−3.635 to −1.042)	−2.679 (−3.635 to −1.042)	−2.603 (−3.300 to −2.275)	0.648
ALBI grade, 1/2/3	223/144/9	214/136/9	9/8/0	0.746
Platelet count, 10^4^/μL	14.4 (3.4–43.1)	14.2 (3.4–43.1)	13.6 (7.9–24.2)	0.483
PT-INR	1.04 (0.88–1.46)	1.04 (0.88–1.46)	1.03 (0.98–1.17)	0.830
Serum AFP, ng/mL	5 (<2–295)	5 (<2–295)	5 (<2–89)	0.423
Serum DCP ^†^, mAU/mL	34 (<30–1280)	34 (<30–1134)	54 (<30–1280)	**0.018**
Device, RFA/MWA	338/38	323/36	15/2	0.686
Concomitant ablation of multiple tumors, yes/no	77/299	71/288	6/11	0.129
Sonazoid-enhancement, yes/no	151/225	141/218	10/7	0.131
Preparing artificial pleural effusion or ascites, yes/no	194/182	184/175	10/7	0.624

Continuous variables are presented as medians (ranges). A bold *p*-value indicates statistical significance (*p* < 0.05). ALBI, albumin–bilirubin; AFP, α-fetoprotein; DCP, des-γ-carboxy prothrombin. MWA, microwave ablation; PT-INR, prothrombin time-international normalized ratio; RFA, radiofrequency ablation. † Serum DCP levels were missing for four treatment courses due to oral warfarin use.

**Table 3:** Factors associated with local tumor progression per tumor.

	Univariate Analysis			Multivariate Analysis		
Variable	HR	95% CI	*p*-Value	HR	95% CI	*p*-Value
Tumor diameter ≥ 20 mm	1.361	0.807–2.293	0.248			
High-risk location	1.689	0.933–3.054	0.084	1.617	0.866–2.826	0.126
Ablation margin of Rvf1′ ^†^	1.844	0.929–3.273	0.083	1.703	0.873–3.074	0.113
Ablation margin of Rvf3 ^‡^	0.542	0.170–1.727	0.300			
During atezolizumab plus bevacizumab treatment	0.121	0.001–0.840	**0.027**	0.124	0.001–0.859	**0.015**

A bold *p*-value indicates statistical significance (*p* < 0.05). CI, confidence interval; HR, hazard ratio. ^†^ Rvf1′ indicates a secured ablative margin except for areas adjacent to blood vessels or bile ducts. ^‡^ Rvf3 indicates a secured ablative margin ≥ 5 mm encompassing the entire tumor.

**Table 4:** Factors associated with local tumor progression per treatment course.

	Univariate Analysis			Multivariate Analysis		
Variable	HR	95% CI	*p*-Value	HR	95% CI	*p*-Value
Age ≥ 65	2.559	0.929–7.054	0.069	2.744	0.993–7.582	0.052
Sex, male	0.972	0.543–1.738	0.923			
Recurrent hepatocellular carcinoma	1.117	0.606–2.059	0.723			
Etiology, non-viral	1.438	0.851–2.430	0.174			
Microwave ablation	0.810	0.325–2.021	0.652			
Child–Pugh grade B	1.820	0.725–4.569	0.202			
Modified ALBI grade 1	0.598	0.318–1.125	0.111			
Serum AFP ≥ 10 ng/mL	2.116	1.251–3.581	**0.005**	2.197	1.280–3.665	**0.005**
Serum DCP ≥ 40 mAU/mL	1.113	0.673–1.839	0.677			
Concomitant ablation of multiple tumors	1.529	0.875–2.672	0.136			
Sonazoid-enhancement	0.682	0.398–1.169	0.164			
Preparing artificial pleural effusion or ascites	0.712	0.433–1.171	0.181			
During atezolizumab plus bevacizumab treatment	0.128	0.001–0.885	**0.032**	0.132	0.001–0.913	**0.037**

A bolded *p*-value indicates statistical significance (*p* < 0.05). ALBI, albumin–bilirubin; AFP, α-fetoprotein; DCP, des-γ-carboxy prothrombin; CI, confidence interval; HR, hazard ratio.

**Table 5:** Ablation procedure-related complications with and without concurrent treatment with atezolizumab plus bevacizumab.

	Concurrent Atezolizumab Plus Bevacizumab Treatment		
Variable	Without (*n* = 359)	With (*n* = 17)	*p*-Value
Total (%)	14 (3.9)	0 (0)	1.000
Portal thrombosis	6	0	
Hepatic infarction	2	0	
Bile duct stricture	2	0	
Formation of biloma	2	0	
Arterial bleeding	1	0	
Ischemic colitis	1	0	

Values are presented as numbers (%).

## Data Availability

The data presented in this study are available from the corresponding author upon reasonable requests.

## Supplementary Materials

The following supporting information can be downloaded at: https://www.mdpi.com/article/10.3390/cancers18111800/s1. Figure S1. Cumulative incidence curves for LTP without censoring marks. Curves represent cumulative incidence estimated using the Kaplan–Meier method (1 − Kaplan–Meier estimates) As censoring marks may obscure the overall trend of the curves, they were omitted in this figure to enhance visual clarity. atezo/bev, atezolizumab plus bevacizumab; LTP, local tumor progression. Table S1. Characteristics of ablated tumors in the ABC conversion and PD salvage subgroups. Table S2. Characteristics of treatment courses in the ABC conversion and PD salvage subgroups.

## Abbreviations

The following abbreviations are used in this manuscript:

ABC conversion	atezolizumab plus bevacizumab curative conversion
AFP	alpha-fetoprotein atezo/bev atezolizumab plus bevacizumab
ALBI	albumin–bilirubin
CI	confidence interval
CR	complete response
CT	computed tomography
DCP	des-gamma-carboxy prothrombin
EOB-MRI	gadolinium–ethoxybenzyl diethylenetriamine pentaacetic acid-enhanced magnetic resonance imaging
HCC	hepatocellular carcinoma
HR	hazard ratio
LRT	locoregional treatment
LTP	local tumor progression
MWA	microwave ablation
PD	progressive disease
RFA	radiofrequency ablation
VEGF	vascular endothelial growth factor
