# Supplementary material for: Reduced Local Tumor Progression After Thermal Ablation During Atezolizumab Plus Bevacizumab Treatment for Hepatocellular Carcinoma
Source: Cancers (Basel). 2026 Jun 1;18(11):1800. doi: 10.3390/cancers18111800 (PMC13255955; doi:10.3390/cancers18111800)
Supplement: Supplementary file 1 [file cancers-18-01800-s001.zip › Figure_S1.pptx]

## Slide 1
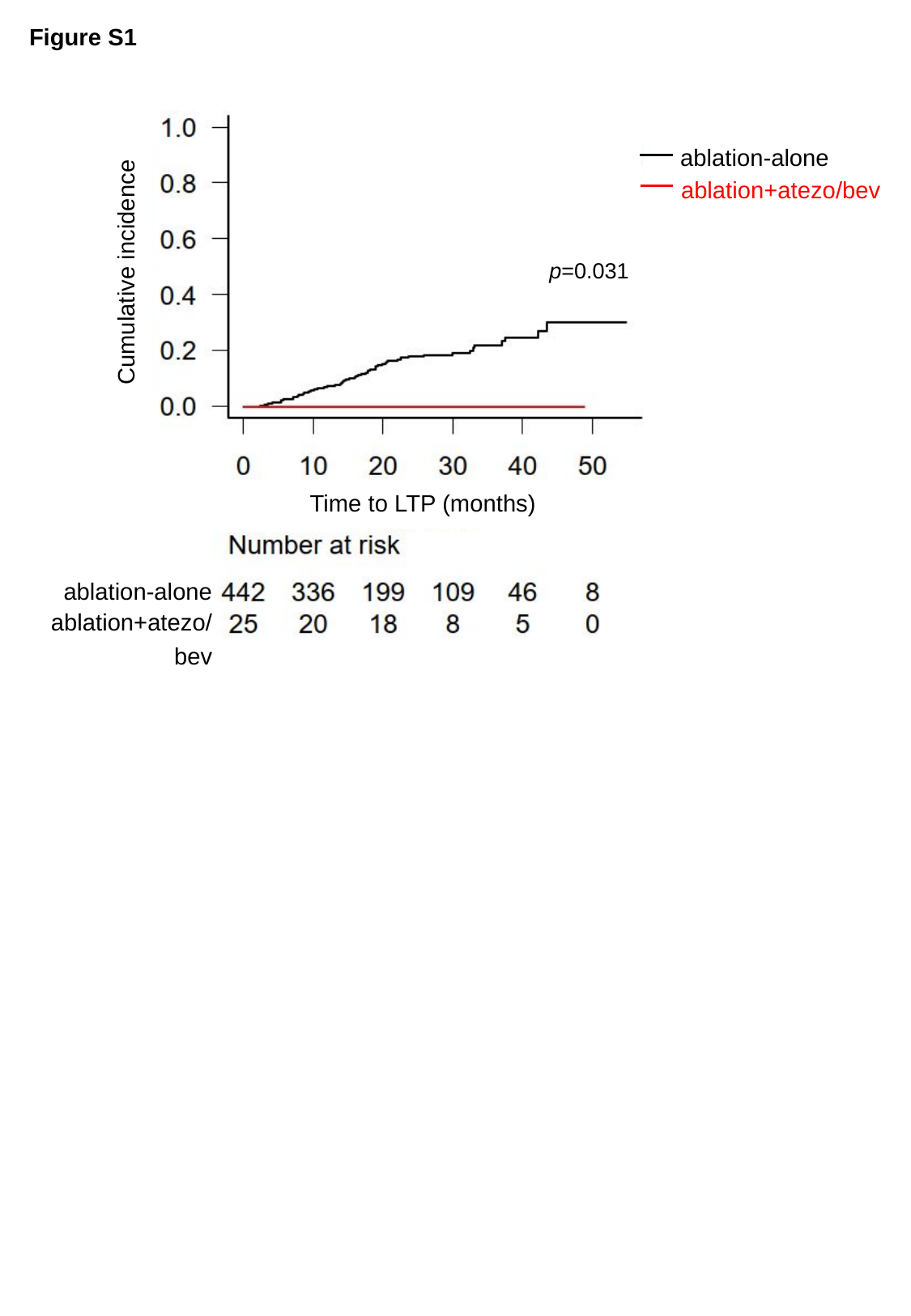

Figure S1
ablation-alone
ablation+atezo/bev
p=0.031
Cumulative incidence
Time to LTP (months)
ablation-alone
ablation+atezo/bev
