# Supplementary material for: Reduced Local Tumor Progression After Thermal Ablation During Atezolizumab Plus Bevacizumab Treatment for Hepatocellular Carcinoma
Source: Cancers (Basel). 2026 Jun 1;18(11):1800. doi: 10.3390/cancers18111800 (PMC13255955; doi:10.3390/cancers18111800)
Supplement: Supplementary file 1 [file cancers-18-01800-s001.zip › Supplementary_Material.pdf]

Supplementary Materials

Reduced local tumor progression after thermal ablation during atezolizumab plus bevacizumab treatment for hepatocellular carcinoma

Tasuku Nakabori*, Kaori Mukai, Taku Miyanaga, Hiroki Takiyama, Keita Sekiya, Takumi Kinomoto, Takanori Masumoto, Jun Murata, Makiko Urabe, Yugo Kai, Ryoji Takada, Minoru Shigekawa, and Kazuyoshi Ohkawa

*** Correspondence:** Tasuku Nakabori, tasuku.nakabori@oici.jp

1 Supplementary Figures and Tables

1.1 Supplementary Figures

Supplementary Figure S1. Cumulative incidence curves for LTP without censoring marks. Curves represent cumulative incidence estimated using the Kaplan–Meier method (1 − Kaplan–Meier estimates) As censoring marks may obscure the overall trend of the curves, they were omitted in this figure to enhance visual clarity.

atezo/bev, atezolizumab plus bevacizumab; LTP, local tumor progression

1.2 Supplementary Tables

Supplementary Table S1

Supplementary Table S2
